# Remote Patient Monitoring Is Scaling Without a Clear Organizational Model: Insights From the Netherlands

**DOI:** 10.2196/101887

**Published:** 2026-05-28

**Authors:** Vincent Peters

**Keywords:** remote patient monitoring, virtual care center, telemonitoring, organizational model, fragmentation

## Abstract

With hospital systems under increasing strain, new digital care models are being rapidly deployed. In this *News and Perspectives* article, JMIR Correspondent and researcher Vincent Peters reports on remote patient monitoring initiatives in the Netherlands and the organizational structures needed for effective implementation.

**Key Takeaways:**

- Remote patient monitoring has emerged as a labor-saving innovation.
- Scaling remote patient monitoring is an organizational challenge, not just a technological one.
- There is no single best organizational model: organizational choices reflect priorities.

*Vincent Peters, PhD, is an assistant professor at the Department of Information Systems and Operations Management at Tilburg University. His research is focused on the digitization of health care, especially on how to organize and integrate remote care with in-person care and how this impacts health care operations and outcomes. In this piece, he highlights the tension between innovations in remote care and the slower evolution of organizational structures needed to support them in the Netherlands, based on his own research and informal conversations with hospital stakeholders.*

Health care systems worldwide are facing growing workforce shortages. The pressure on health care professionals continues to increase, while waiting times for patients are rising. These shortages are projected to worsen globally in the coming years. As the chief medical information officer of a Dutch hospital described it, the situation resembles “A car speeding toward a stationary wall requiring action from those behind the wheel.” Clearly, labor-saving innovations are no longer a choice but a necessity. Remote patient monitoring (RPM) has emerged as one such innovation.

## The Rise of Remote Patient Monitoring

Over the past decade, RPM has increasingly complemented traditional in-person care, enabling patients to be monitored in their homes while health care professionals oversee their condition remotely. The COVID-19 pandemic accelerated RPM adoption at an unprecedented pace, and importantly, this shift has proven durable rather than temporary. To date, many global health care systems maintain or expand their RPM offerings in the postpandemic period.

**Figure:**
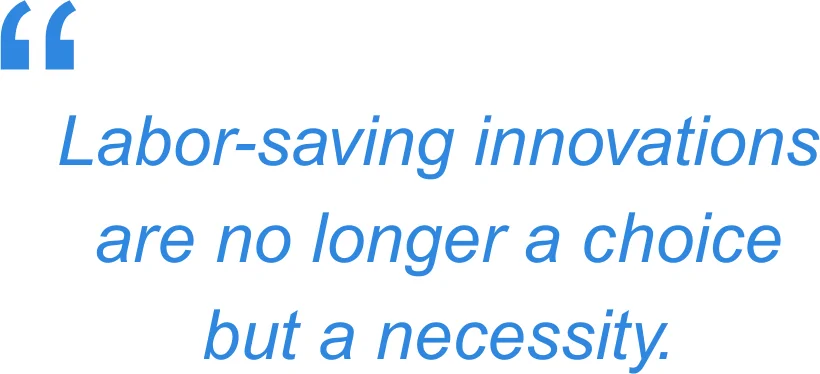


However, despite its rapid expansion, there is no shared understanding of how the organizational structures underpinning RPM should be designed, governed, or embedded within hospital operations. In practice, this leads to fragmented approaches, duplication of efforts, and missed opportunities for learning across hospitals. As the head of a centralized Dutch virtual care center mentioned, “We organize it [RPM] in this way, because we believe in it, not because we have evidence for it,” indicating that they just went for it. In many cases, RPM is done alongside and on top of regular work, limiting its true potential to generate meaningful outcomes.

This ambiguity is also reflected in the literature. While there is growing evidence on the clinical effectiveness of RPM, far less attention has been paid to its organizational design. In the absence of clear guidance, decisions are often made based on local clinical or administrative judgment rather than evidence-based approaches. As a result, hospitals are left to define their own organizational solutions.

## Emerging Organizational Models

In the absence of a shared understanding, four organizational models for organizing RPM have emerged in Dutch practice. These models differ in where monitoring activities take place, how responsibilities are distributed, the clinical proximity to the patient, and the extent to which care is outsourced.

### Decentralized Within the Hospital

In this organizational model, RPM is organized at clinical departments within the hospital, in which health care professionals monitor their specific patient population. This more traditional approach is often referred to as telemonitoring. RPM activities are added to day-to-day practices, offering clinical integration and continuity of care. Rather than outsourcing to dedicated telenurses, these activities are managed by professionals embedded within the specific clinical department, who are already familiar with the patient’s history and treatment context. It can also support professional development and ownership among staff. However, since RPM is performed alongside regular duties, 24-7 coverage is difficult and creates dependency on a small number of professionals.

### Centralized Within the Hospital

Here, RPM activities are consolidated into a single hospital-wide unit. This so-called virtual care center (or medical service center) is an organizational entity that uses digital technologies to remotely monitor patients, interpret incoming patient health data, and deliver and coordinate medical care within the patient’s home environment. This unit allows for monitoring across specialties and is handled by a dedicated team, allowing for greater standardization and potentially faster response times. This centralized model within the hospital can improve efficiency, enable workforce specialization, and create the base for scaling RPM across the hospital.

The same centralized virtual care center head illustrated, “Our virtual care center allowed us to scale up remote patient monitoring in ways that we could have never imagined. We started small with two pilots and now offer more than 50 care pathways in a remote way...and these numbers ensure that we can staff our center and expand our opening hours to serve our patients in the best possible way.”

However, this model requires coordination and up-front investment, including new workflows, new roles for those who staff the virtual care center like telenurses, and physical/virtual infrastructures. Clinical distance from clinical departments may also pose challenges, and continuous monitoring is not always feasible without additional arrangements.

### Outsourced Centralized Model

In this model, all RPM activities are managed by an external virtual care center, which monitors patients on behalf of one or more hospitals. This model has the potential for standardization of care pathways, combined with centralized coordination and digital infrastructure, allowing monitoring activities to be organized more uniformly across patient groups. However, outsourcing reduces direct control and increases clinical distance to the patient. Telenurses staffing the external virtual care center might not be aware of the patient’s local context, which can affect how signals are interpreted.

As the project manager of a Dutch outsourced centralized model noted, tongue-in-cheek, patient behavior and symptom reporting can vary significantly by region: “You hear differences in the Netherlands, because in Groningen, when someone is ‘not feeling well,’ they are lying half-dead on the couch. While in Amsterdam, ‘not feeling well’ means immediately going to the hospital.” Without this contextual familiarity, there is a risk of misinterpretation or overstandardization. In addition, this outsourced model creates dependency on external partners and requires agreements on data sharing, clinical responsibility, and quality assurance.

### Hybrid Model

In a hybrid model, hospitals retain responsibility for RPM in some care pathways, while outsourcing others to an external virtual care center. Outsourcing is typically done for pathways that require continuous or high-intensity monitoring, such as acute or perioperative monitoring. This model allows for greater flexibility in how RPM is organized, for example, by tailoring the approach per care pathway. It can help address capacity constraints by shifting workload but also increases complexity and requires clear agreements on responsibilities and data exchange.

## Choosing the “Right” Model

Picking the right organizational model for RPM is not an easy task. Different models reflect different priorities, and no single model optimizes all outcomes. Hospitals may prioritize differing outcomes, and models support these aims differently. For example, decentralized models may strengthen professional ownership and continuity, while centralized or outsourced models may favor efficiency and scale.

At the same time, choices are shaped by contextual factors, including hospital size and available resources, the characteristics of the patient population, and the nature of care pathways. For instance, stable, low-complexity conditions may not require close clinical proximity, whereas more complex cases may benefit from stronger integration within clinical departments. Geographic context also plays a role, as regional density and opportunities for collaboration influence whether RPM can be organized locally or across hospitals.

**Figure:**
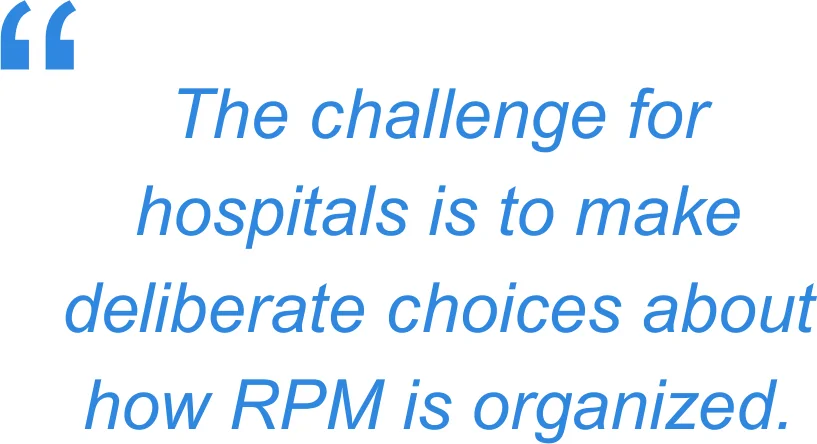


Hence, the key question is not which model is objectively *best*. Nor should RPM be treated as a goal in itself. Rather, the challenge for hospitals is to make deliberate choices about how RPM is organized. The four models outlined here provide a practical lens to structure these choices and make trade-offs explicit. Different priorities will naturally lead to different models, and there is no one-size-fits-all solution. However, this makes it all the more important to adopt a structured approach, rather than relying on ad hoc decisions. Otherwise, RPM risks remaining stuck in experimentation and fragmentation.

